# What speech and language therapy do community dwelling stroke survivors with aphasia receive in the UK?

**DOI:** 10.1371/journal.pone.0200096

**Published:** 2018-07-10

**Authors:** Rebecca Palmer, Helen Witts, Timothy Chater

**Affiliations:** 1 School of Health and Related Research, University of Sheffield, Sheffield, United Kingdom; 2 Speech and Language Therapy, Derbyshire Community Health Services NHS Foundation Trust, Chesterfield, United Kingdom; Kobenhavns Universitet, DENMARK

## Abstract

**Background:**

Speech and language therapy provision for aphasia (a language disorder) post stroke has been studied over time through surveys completed by speech and language therapists. This paper revisits provision based on what was received by 278 patients in 21 UK speech and language therapy departments in 2014–2016.

**Aims:**

To explore the speech and language therapy received by community dwelling people with post stroke aphasia in the UK.

**Methods and procedures:**

A quantitative content analysis was conducted by two speech and language therapist researchers. Therapy goals recorded were coded into categories and subcategories. Descriptive statistics were used to identify the frequency with which goal categories were targeted, average therapy time received, length and frequency of therapy sessions, personnel involved and mode of delivery.

**Outcomes and results:**

Forty-five percent of participants were in receipt of therapy in the three month window observed. Six goal categories were identified. Rehabilitation was the most frequent (60%) followed by enabling (17.2%), review (4.3%), assessment (3.6%), supportive (3.5%) and activity to support therapy (2.8%). The median amount of therapy received in three months was 6.3 hours at an average of one 60-minute session every two weeks. Seventy-seven percent of therapy sessions were delivered by qualified speech and language therapists and 23% by assistants. Ninety percent of sessions were one to one, face to face sessions whilst 9.5% were group sessions.

**Discussion:**

In line with previous reports, speech and language therapy for community dwelling stroke survivors with aphasia is restricted. Rehabilitation is a large focus of therapy but the intensity and dose with which it is provided is substantially lower than that required for an effective outcome. Despite this, one to one face to face therapy is favoured. More efficient methods to support more therapeutic doses of therapy are not commonly used in routine clinical services.

## Introduction

Aphasia is an acquired language disorder affecting a person’s spoken comprehension, ability to express themselves using spoken language, and their ability to read and to write. One third of people acquire aphasia as a consequence of stroke [1]. Speech and language therapists are trained to provide intervention to reduce the extent and impact of the language impairment. The purpose of this paper is to explore the speech and language therapy that is received by community dwelling people with post stroke aphasia across NHS trusts in the UK (2014–2016).

The goals people with aphasia wish to achieve have been explored by Worrall et al (2010) by interviewing 50 people living with aphasia [2]. The main goals identified included returning to pre stroke life; communicating opinions as well as basic needs; being provided with information about aphasia, stroke and services available; wanting more speech therapy, greater autonomy, dignity and respect; being able to engage in social, leisure and work activities; regaining physical health and helping others. These were mapped onto the World Health Organisation International classification of functioning, disability and health demonstrating that people with aphasia have goals that span the spectrum of body function and structures (the impairment); activity (enabling greater use of language); participation (usual activity) and environmental factors [3]. Speech and language therapy is integral to addressing these goals and Malcomess (2005) developed a framework of Care Aims for speech and language therapists to reflect on the purpose of their intervention, categorised into investigation (assessment); prevention; stabilisation (maintenance); participation (enabling); resolution (curative); improvement (rehabilitation); adjustment (supportive) and comfort (palliative) [4].

Helping people with aphasia to achieve their goals can be addressed by improving the language impairment itself which involves rehabilitation of the body function/structures causing the impairment; reducing the impact of, or compensating for, the language impairment by enabling the individual, communication partners and the communication environment to adopt supplementary or compensatory strategies to communicate and thus participate in usual activities; supporting individuals and families with emotional consequences of changes in their communication. Therapy time is required to practise improving language ability and to practise and adjust to using new ways of communicating. Traditionally it has been thought that recovery from language can reach a ‘plateau’ which has led to intervention not being offered if a patient is more than 6 months or a year post stroke in many places. However, there is evidence to suggest people can improve their communication with therapy in the chronic phase. In a review of 21 RCTs, Allen et al 2012 found evidence to support the use of computer based treatments, constraint induced language therapy, group therapies and training of communication partners more than 6 months post stroke [5]. Time post onset is not thought to be related to response to treatment in chronic aphasia [6]. The Cochrane review of speech and language therapy for aphasia following stroke included 57 randomised controlled trials (RCTs) of aphasia therapy [7]. Results of 27 RCTs demonstrated that SLT led to clinically and statistically significant benefits to patients’ functional communication, reading, writing and expressive language. Analysis of RCTs that compared quantities of therapy delivered showed that functional communication was significantly improved in people with aphasia who received therapy at high intensity (from 4 to 15 hours a week), high dose (27–129 hours in total) or over a long period of time (up to 22 months) compared with those who received lower intensity or dose treatments over shorter periods of time. These findings support the results of Bhogal et al’s (2003) analysis of RCT data that showed total therapy time correlated with improvement [8]. A recent RCT of 158 people with aphasia in the chronic phase (more than 6 months post stroke) again demonstrated the benefits of high intensity therapy (10 hours or more over three weeks) compared with a phase of no therapy for a group of participants on waiting lists for therapy [9]. Similar evidence for high intensity, high dose, long duration of therapy is echoed across the wider rehabilitation community for stroke (e.g. OT, physiotherapy) resulting in Royal College of Physicians (RCP) clinical stroke guidelines that recommend provision of at least 45 minutes of therapy a day for as long as a person is benefitting from it [10].

Therapy provision for aphasia has been studied at intervals spanning the last 25 years. In 1993 MacKenzie et al published a survey of 90% of National Health Service (NHS) speech and language therapy services in the UK [11]. Only 25% of therapists were able to treat community dwelling patients with aphasia at least three times per week with the most likely frequency of treatment being offered between one and three times a week. In 1993 53% of UK SLTs were able to continue to treat patients for more than a year whilst 17% could treat for a maximum of six months and 10% for a maximum of three months. Code and Herron (2003) conducted a similar survey of 74 NHS SLT departments in the UK almost a decade later in 2000 [12]. The intervening years saw double the number of SLTs working with neurological communication disorders, although a rise in dysphagia referrals (swallowing difficulties) to this same staff group meant only 26% of SLTs’ time was spent working with aphasia and of this time, only 4.8% was spent in providing therapy sessions of more than three hours a week. Katz et al (2000) conducted an international survey of SLTs providing services for adult neurological communication disorders in Australia, Canada, UK, US private sector and US Veterans Affairs [13]. Services to community dwelling people with communication disorders was limited across all the nations surveyed with the maximum amount of therapy being offered by the US private sector (mean nine sessions; range one to 20 sessions). Verna et al (2009) added to this information for Australia with a survey showing that people living with chronic aphasia in the community received just one hour of SLT a week on average [14]. The amounts of speech and language therapy available to people with aphasia in practice have therefore consistently fallen short of the intensity, duration and dose shown to be required for effective therapy in the literature. In 2013, Rose et al conducted a survey of 188 SLTs in Australia and reported inflexible funding models as major barriers to providing care and lack of resources as a challenge to effective service provision [15]. Ways to increase therapy provision to the recommended levels without increasing the need for additional therapist time has been considered in the literature, for example providing therapy in groups, or training volunteers and assistants and using specialist computer software and apps to support the repetitive practice of tasks to promote language improvement, and to practise conversation and functional communication skills [16, 17, 18, 19].

International and UK surveys of SLT practices for community dwelling stroke survivors living with aphasia have predominantly focussed on the amounts of intervention provided for the population with aphasia as a whole, with two surveys also identifying the focus or mode of delivery of intervention SLTs provide in Australia [14, 15]. Both papers found a functional or social approach to therapy is most frequently taken. Verna et al (2009) identified that one to one therapy was the predominant mode of delivery across the continuum from acute to community and Rose et al (2013) found that group treatments were rated as less appropriate than one to one [14, 15]. The Big CACTUS randomised controlled trial [19] took place in 21 National Health Service (NHS) speech and language therapy (SLT) departments across the UK with patients with aphasia in the community randomised to usual care alone, usual care plus computer therapy, or usual care plus attention control. Usual care was recorded for all 278 participants providing a unique opportunity to observe current (2014–2016) SLT provision in community settings across the UK, to update the information on the amount of intervention provided and introduce new information on the therapy goals recorded by SLTs in the UK, and the mode of therapy delivery. This study is also unique in its review of therapy provision from the perspective of what individual patients received, rather than surveys of therapist perspectives on what they provide.

The key questions asked are: 1) Who receives speech and language therapy in the community? 2) What are the goals of therapy? 3) How much therapy is received? 4) Who delivers therapy and how is it delivered?

Each of these questions is explored in the paper in relation to the community dwelling population with aphasia as a whole but also adds a further dimension to the current literature by answering these questions for subgroups of people with aphasia according to time post stroke, severity, and age.

## Methods and materials

### Design

A quantitative content analysis was used to identify and analyse therapy goals recorded for 278 people with aphasia, over a period of three months prior to randomisation to the Big CACTUS trial. Analysis followed the six stages of content analysis proposed by List (2007): 1) selecting content for analysis, 2) identifying the units of content, 3) preparing content for coding, 4) coding content, 5) counting and weighting, 6) drawing conclusions [20]. Descriptive statistics were used to explore who receives therapy, the frequency of different types of therapy goals, the amount of therapy received, who delivers therapy and how.

### Participants

People with aphasia post stroke were recruited to the Big CACTUS trial from 21 NHS trusts across the UK [19]. Potential participants were identified by speech and language therapists working in the NHS from current and past patient case-loads, and from local voluntary support groups. Inclusion criteria for the trial were as follows: aged over 18 years; had a diagnosis of stroke at least 4 months prior to randomisation; had a diagnosis of aphasia subsequent to stroke; were able to retrieve 10–90% of words on the Comprehensive Aphasia Test Naming Objects subtest [21]; were able to perform a simple matching task in the StepbyStep [22] computer programme with at least 50% accuracy and were able to repeat at least 50% of words in a StepbyStep [22] repetition task. Participants were excluded if they had another pre-morbid speech and language disorder caused by a neurological deficit other than stroke; required treatment for a language other than English (as the computer program was in English only); were already using the StepbyStep [22] computer program or other computer speech therapy aimed at word retrieval/naming at the time of recruitment to the trial.

Recruitment was carried out by speech and language therapists trained by the Big CACTUS research team. The Consent Support Tool [23] was used to ensure informed consent was taken where possible and when a potential participant did not demonstrate capacity to consent, their inclusion was enabled by a signed consultee declaration by a relative of belief that they would like to participate. The word finding severity of each participant was calculated following assessment with the Object Naming test of the Comprehensive Aphasia Test [21]. Those that scored 31 to 43 out of 48 were classed as mild; 18 to 30 out of 48 as moderate and 5 to 17 out of 48 as severe. See Big CACTUS protocol [19] for further details. The study protocol was approved by Leeds West NHS research ethics committee [reference 13/YH/0377]. Additional approval was granted for Scotland by the Scotland A research ethics committee [reference 14/SS/0023].

### Procedure for recording usual care provided by speech and language therapy

The lead therapist for the Big CACTUS study in each NHS trust completed an ‘information about usual care’ form at baseline (after the participants had been consented but before they were randomised to a study intervention arm). This data was entered on the Big CACTUS trial data management system. The form prompted the lead therapist to find out whether the participant had received any care for their communication difficulties in the past three months. At baseline, care was reported for the three-month time period before randomisation. The participant and or carer was asked whether they had received any speech and language therapy in the last three months. Information reported by the participant was then supplemented by searching electronic databases, patient records and speaking to speech and language therapy colleagues who may have delivered the interventions in clinical practice. The ‘information about usual care’ form and electronic data management system prompted the recording of the date of therapy, the agenda for change (AfC) band of the clinician or assistant delivering the therapy (AfC is the grading system used for all NHS therapists in the UK), the goal(s) of the therapy session, the duration of the therapy session, the mode of therapy delivery (indirect: telehealth, video call, telephone; face to face: one to one, group), and the distance that was travelled if therapy was delivered face to face. Incomplete or missing data was checked by the trial data managers at regular intervals and discrepancy reports were produced and sent to lead therapists in each NHS trust to check the information or confirm that it was not available.

### Content analysis procedure

The purpose of the content analysis was to identify types of therapy goals recorded. The analysis was undertaken by two speech and language therapy researchers experienced in delivering aphasia therapy and familiar with how the data was collected in the Big CACTUS study (authors RP and HW).

Selection of content for analysis: Therapy goals collected at the baseline time point were selected for analysis. These goals were those being worked towards in therapy sessions that took place in the three months before a participant was randomised to the computer therapy trial enabling observations of therapy received without potential influences of participation in the trial as trial intervention had not yet begun. As participants had to be a minimum of four months post stroke at randomisation, the retrospective observation of therapy received over the previous three months includes observations from one month post stroke.

Units of content: Therapy goals were recorded for each therapy session received. Some therapy sessions addressed more than one therapy goal. The unit of content used for analysis was each individual goal.

Preparing the content for coding: Each therapy session reported one or more goals in the treating therapists’ own words. Firstly, each goal within a session was identified. Where multiple goals were identified, the session was split according to the number of goals in that session. Secondly, the goals were checked by all authors of this paper to identify goals that were essentially the same by correcting spelling errors/typos and introducing consistency in terms of capitalisation, hyphenation and spaces. Consistency was also introduced where it was clear to the speech and language therapists analysing the data that goals were synonymous e.g. ‘goal set’, ‘goal setting’ and ‘set goals’. The authors agreed that it was important to analyse these data remaining true to the descriptions of goals used by therapists and therefore even when goals were similar e.g. ‘word finding’ and ‘naming’ the original wording was retained. This process resulted in a list of unique goals for analysis. (See S2 File)

Coding the content: The unique goals were coded by the two speech and language therapist researchers (authors RP and HW). A coding framework was developed with reference to the Malcomess (2005) care aims [4]. The framework was developed iteratively, starting with the first author (RP) reviewing the first 200 unique goals and identifying them according to care aims e.g. rehabilitation, enabling, supportive, assessment, maintenance by comparing the goal with the care aim descriptors [4]. Where a goal did not appear to be well described by a care aim, additional codes were added to the framework to encompass these goals. Within each high level code, goals were also coded into more specific descriptions of the type of goal described e.g. ‘enabling: participation in social conversation/activities’. By the 200^th^ goal, no additional codes needed to be created. The coding framework was sense checked by the second author (HW) who then used it to independently code the same 200 goals. The two coders were in 95% agreement on the high level codes and 94% agreement on the second level codes. The coders discussed the discrepancies which resulted from either different interpretations of the code descriptions or the presence of very similar code descriptions which could be confused. The coders agreed new definitions for the codes to aid consistent interpretation and amalgamated codes that were very similar. The final coding framework was applied to all 359 unique goals independently by the two coders with reliability scores of 92% and 88% for high level and second level codes respectively. Where coding differed between coders, these goals were discussed and an agreement for their coding was reached. The final codes were added to the file of unique goals (see S2 File). The codes were then mapped to the file recording each unique goal in each therapy session for every participant (S3 File).

Counting and weighting: The coding framework was used to provide a descriptive summary of the types of therapy goals addressed and descriptive statistics were applied to identify the frequency with which different types of therapy were provided.

In addition, the length of therapy session, number of sessions, the Agenda for Change band of therapist or therapy assistant/volunteer delivering the therapy in the session, and the mode of delivery was added to S3 File and again descriptive statistics were used to identify who receives therapy, how much therapy is received, who delivers it and how it is delivered.

S1 File details the participants’ age, length of time post stroke, aphasia severity, and location (NHS trust participating in Big CACTUS). As these details were calculated initially at the point of randomisation to the trial, the ages of the participants three months prior to randomisation and length of time post stroke three months prior to randomisation were calculated and added to S1 File to ensure age and time post stroke were consistent with the three-month window from which data was being analysed for this paper. Using these demographic data, the same analysis of type, and quantity of therapy was performed for subgroups of age three months prior to randomisation (18–34, 35–44, 45–54, 55–64, 65–74, 75–84, 85+); gender; length of time post stroke three months prior to randomisation (1–3 months, 3–6 months, 6–12 months, 1–2 years, 2–5 years, 5+ years); aphasia severity (mild, moderate, severe) and location (Big CACTUS sites).

## Results

### Participants

**Fig 1:**
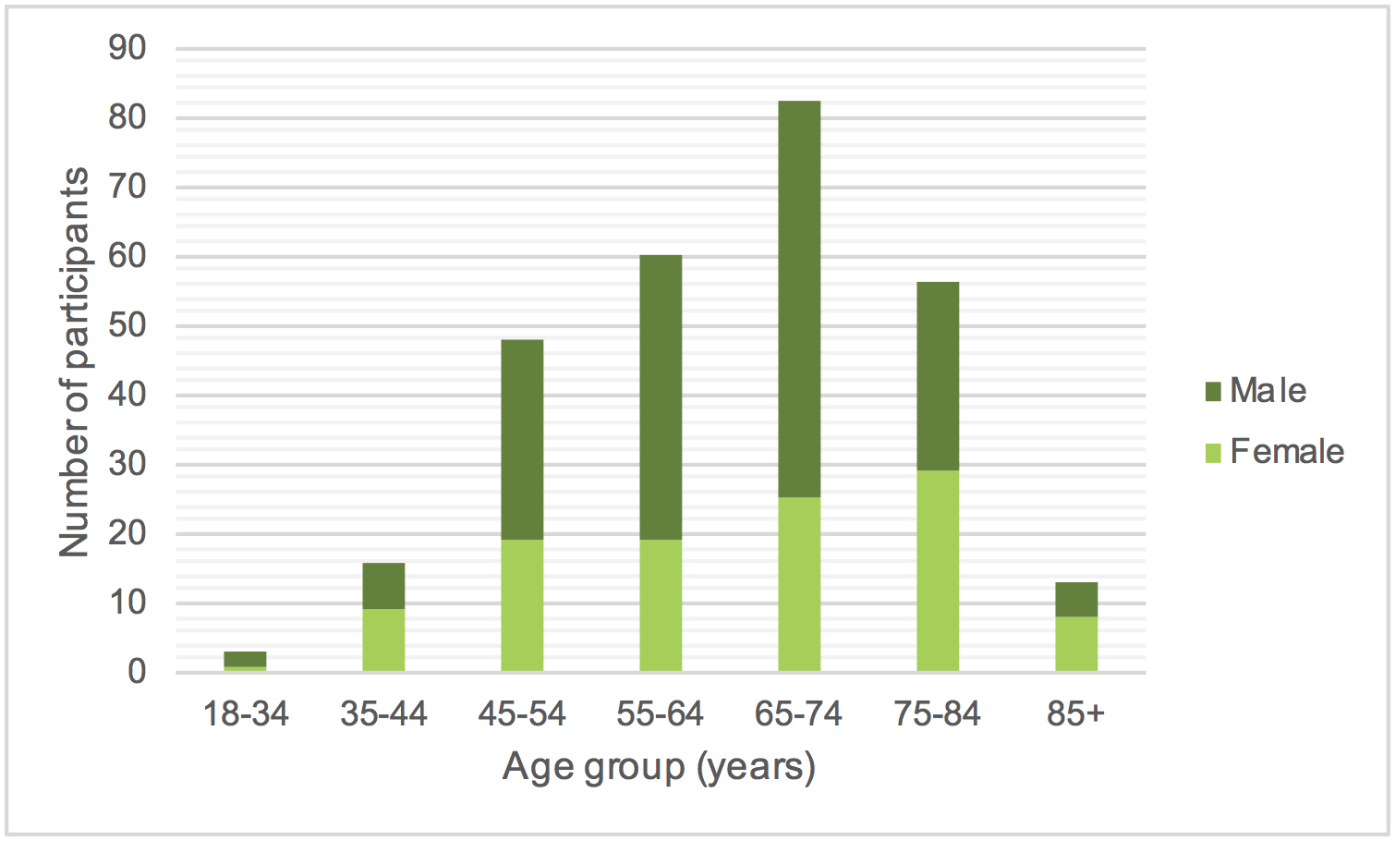
Distribution of participants by age and gender.

**Fig 2:**
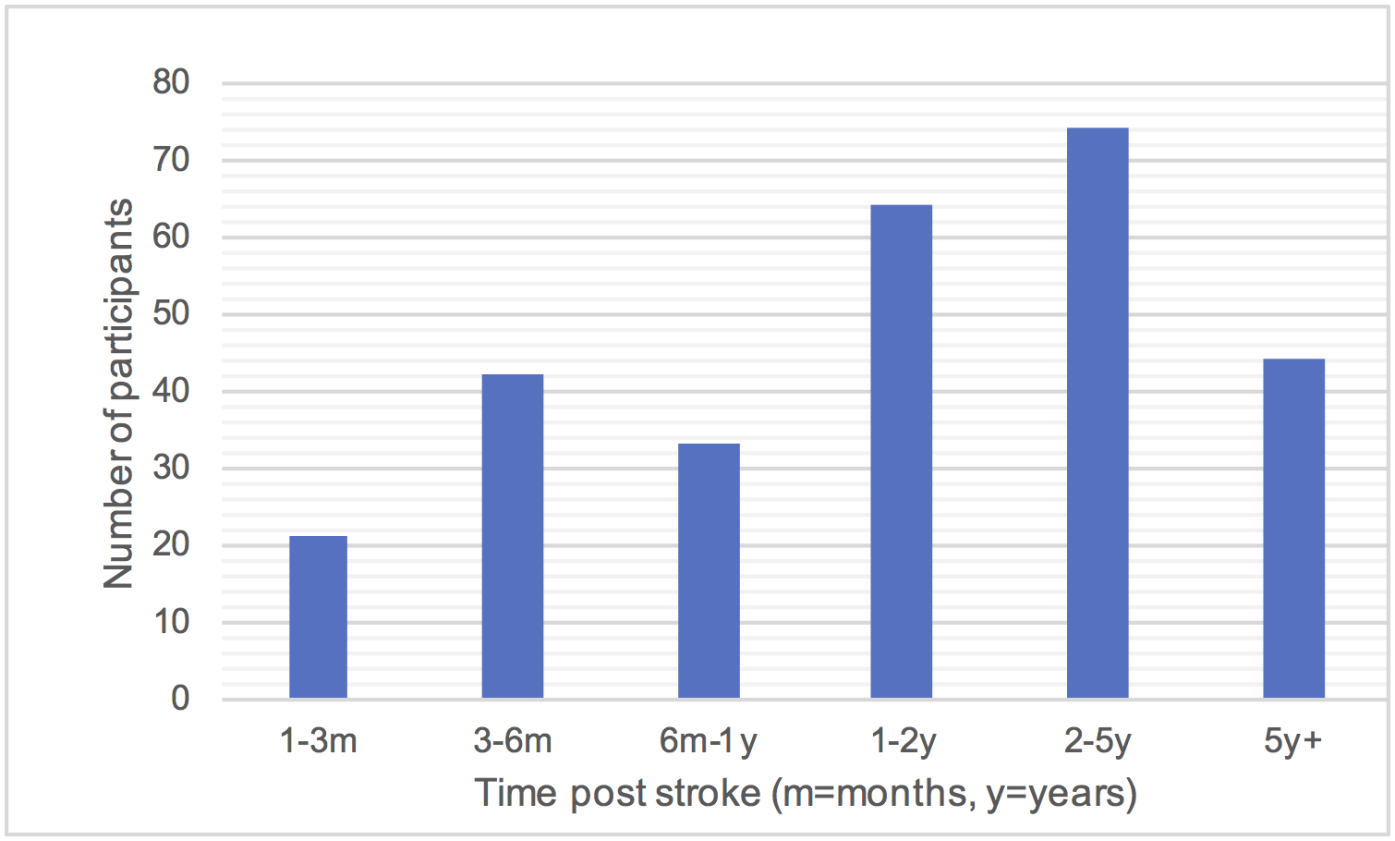
Distribution of participants by length of time post stroke (at the beginning of the three month period analysed).

Of 278 participants with usual care data recorded three months prior to randomisation into the Big CACTUS study, 168 were male and 110 were female with a mean age of 64.9 years and age range 22 to 91 years. Participants ranged from 1 month to 36 years post stroke. Fig 1 shows the distribution of participants by age and gender and Fig 2 shows the distribution of time post stroke. The severity of aphasia of the participants was as follows: 123 (44.3%) mild, 81 (29.1%) moderate and 74 (26.6%) severe. Table 1 shows the number of participants from each participating NHS trust showing the information about usual care is representative of a range of NHS trusts across the UK. These include trusts in England, Wales, Scotland and Northern Ireland.

**Table 1:** Number of participants from each participating NHS trust.

NHS trust	UK Nation	Number of participants	Rural/urban area
A	Scotland	9	Rural
B	Ireland	11	Urban
C	England	13	Rural
D	Wales	9	Rural
E	England	16	Rural
F	England	12	Rural
G	Scotland	22	Urban
H	England	15	Mixed rural/urban
I	England	15	Urban
J	England	10	Rural
K	England	11	Mixed rural/urban
L	England	11	Rural
M	England	15	Mixed rural/urban
N	Ireland	13	Mixed rural/urban
O	England	19	Mixed rural/urban
P	England	8	Rural
Q	England	15	Urban
R	England	16	Rural
S	England	16	Mixed rural/urban
T	England	10	Mixed rural/urban
U	Wales	12	Mixed rural/urban

### 1) Who receives therapy in the community?

**Fig 3:**
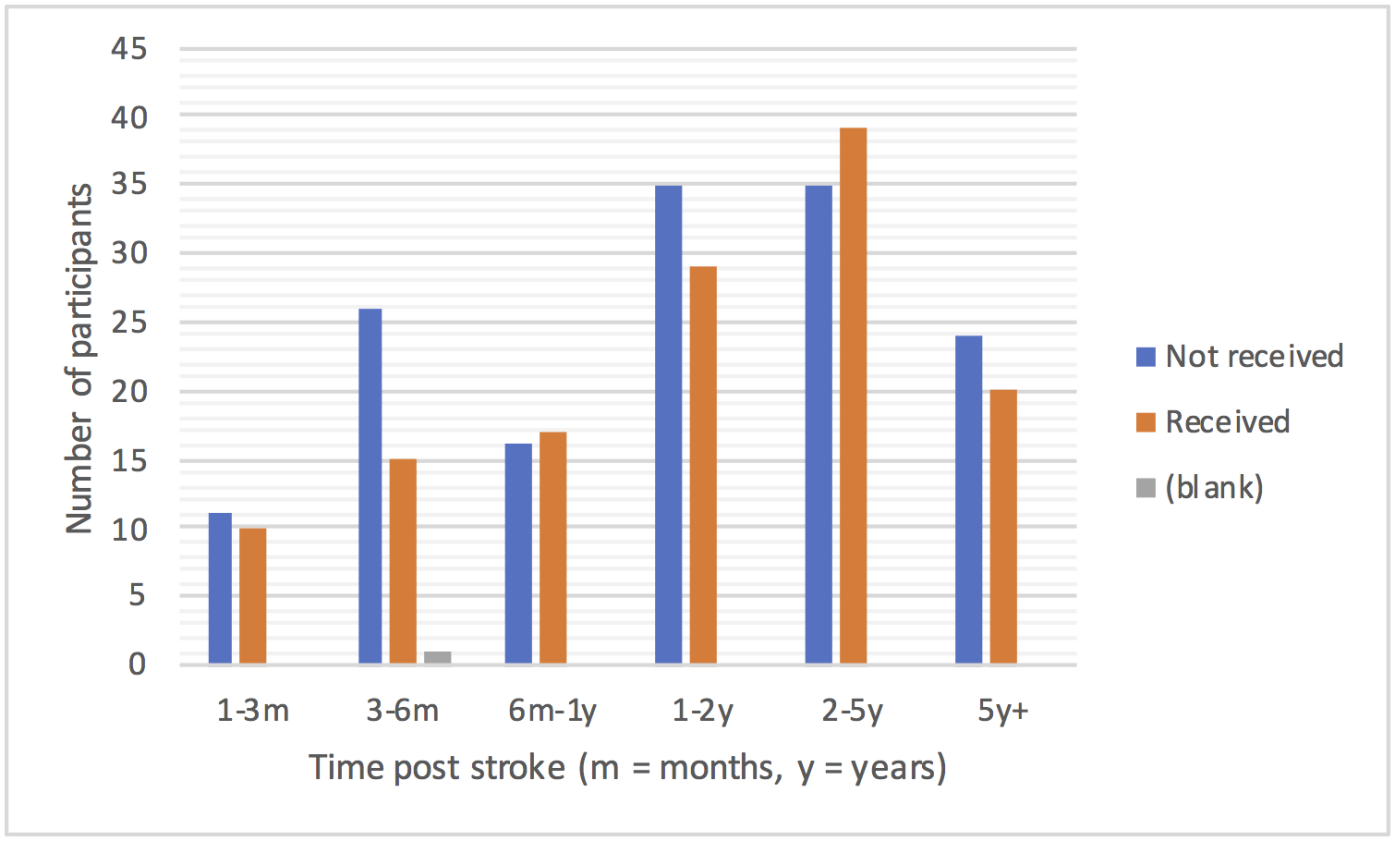
Receipt of therapy by time post stroke.

**Fig 4:**
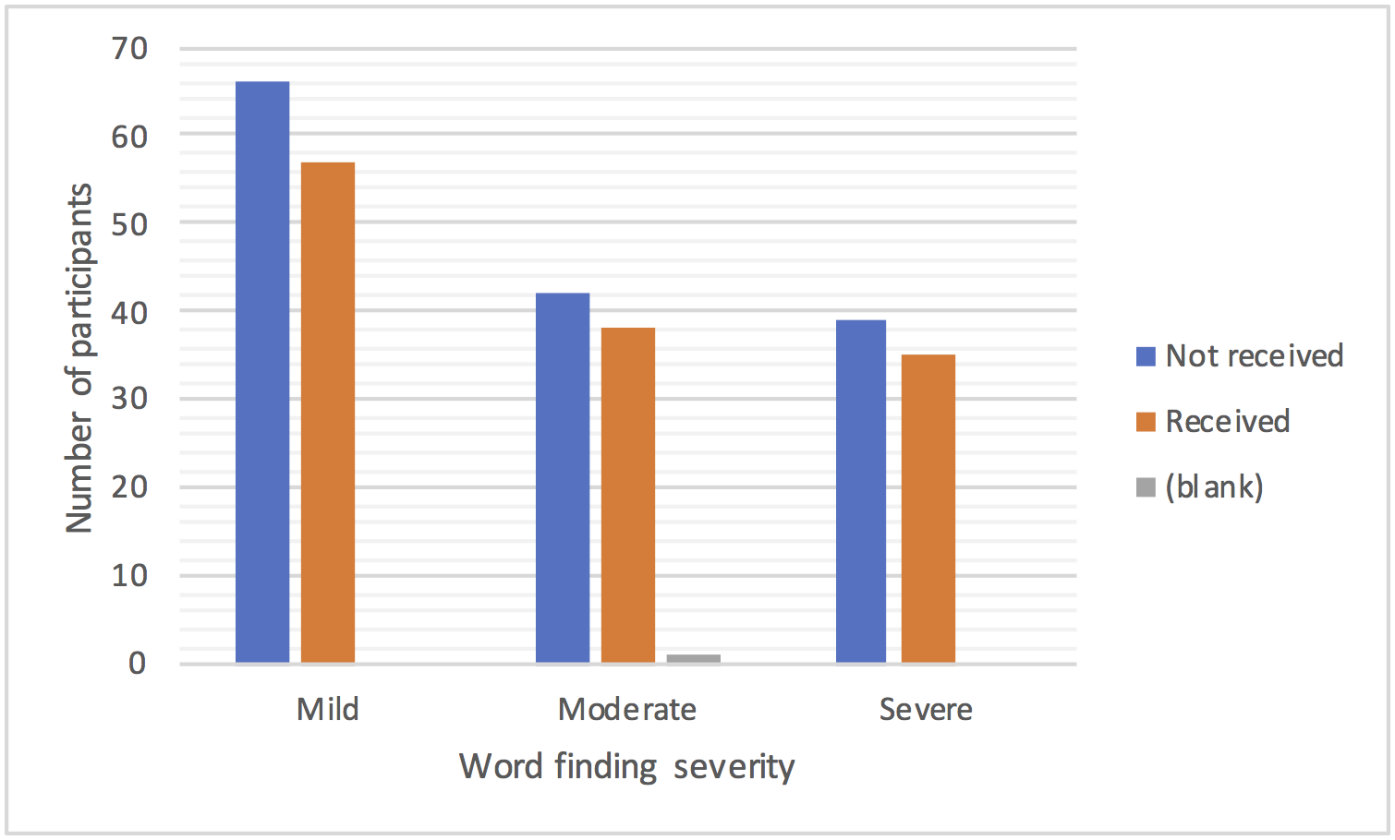
Receipt of therapy by severity of word finding.

**Fig 5:**
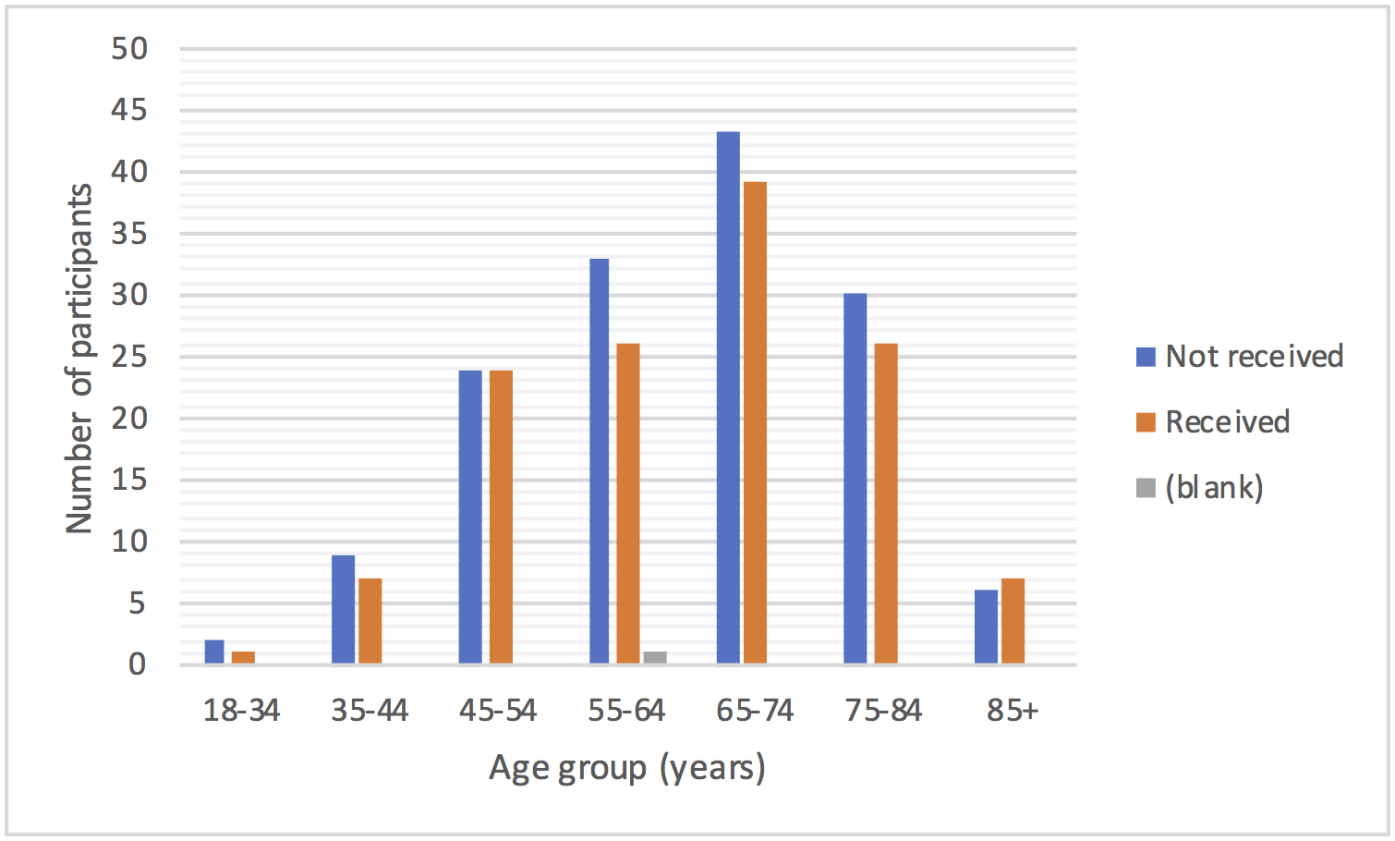
Receipt of therapy by age.

In the three months prior to randomisation to the Big CACTUS study 125(45%) of the 278 participants were in receipt of speech and language therapy. Figs 3–5 show the numbers of participants receiving or not receiving therapy in that three-month window by time post stroke and severity of aphasia. Fig 3 shows that more participants received therapy than did not receive therapy in the first 12 months post stroke, with the pattern switching to it being more likely not to receive than to receive therapy after one year. Fig 4 shows that similar numbers of participants with moderate and severe aphasia did and did not receive therapy, with one and a half times as many people with mild aphasia not receiving therapy as receiving it. Fig 5 does not show any clear effect of age on the receipt of therapy and the small numbers of participants in the youngest and oldest age groups make it difficult to draw conclusions. Table 2 shows the proportion of participants receiving therapy from each NHS Trust ranges from 20% to 91%. Given that Fig 3 suggests that therapy is less likely to be received one year or more after stroke, Table 2 shows the percentage of participants that were more than one year post stroke in each Trust. In seven of the Trusts (A, B, E, M, R, S, T) the percentage of participants not receiving therapy was within 10% of the percentage of participants more than one year post stroke, suggesting time post stroke may be the reason for not receiving therapy in these Trusts. However, for sites C, I, J, K and Q the proportion of participants not in receipt of therapy is a minimum of 20% less than the percentage of participants more than one year post stroke suggesting that participants often receive therapy more than one year post stroke in these NHS Trusts. Conversely, other Trusts such Trust U were not providing therapy to far more participants than were more than a year post stroke, suggesting the presence of other factors limiting provision of therapy at those sites.

**Table 2:** Percentage of participants in receipt of therapy at each participating NHS trust.

NHS trust	Participants receiving therapy (%)	Participants not receiving therapy (%)	% more than 1 year post stroke
A	78	22	22
B	18	82	91
C	54	46	85
D	56	44	78
E	31	69	75
F	59	41	67
G	41	59	77
H	53	47	60
I	47	53	73
J	60	40	60
K	91	9	55
L	27	73	55
M	33	67	60
N	23	77	62
O	63	37	68
P	37.5	62.5	75
Q	47	53	73
R	50	50	56
S	31	69	62.5
T (no information given for 10% of participants at this site)	20	70	70
U	33	67	33

### 2) What are the goals of therapy?

The examples in Table 3 show that some goals were expressed as goals for the patient e.g. ‘to be able to find words in conversation with more ease’, whilst others were goals to be achieved by the therapist e.g. ‘to advise patient and family about the impact and recovery from aphasia’. Other goals were expressed as the focus of the therapy session e.g. ‘money handling skills’ rather than as a specific goal to be achieved. See S2 File for the complete list of unique goals (NB these data were collected retrospectively from patient notes suggesting that not all therapy sessions had specific goals associated with them, however, in all but 8.56% of cases, the activity focus of the session was sufficient to identify the likely goal category and description. Table 3 shows that there was insufficient information to identify goals in 8.56% of sessions, either because the goal or activity was not recorded or not sufficiently described. 60% of the goals for community dwelling patients with aphasia were rehabilitation goals, described by Kate Malcomess as being aimed at reducing the problem or improving skills [4]. Expressive language, word finding and reading received the most attention. The next largest goal category was ‘enabling’ at 17.23%. Kate Malcomess describes ‘enabling’ as reducing the impact of the problem and increasing functioning so that the person can take part ore in his/her daily life [4]. The most frequent enabling goal described was that of conversation support. Many of the other enabling goals focussed on learning strategies to reduce the impact of the language impairment. A much smaller number of therapy goals (3.46%) were focussed on being ‘supportive’ for which Malcomess also uses the term ‘adjustment’ and describes as facilitating changes in feelings, attitudes and insights about the presenting problem [4]. One of the largest supportive roles taken by SLTs was increasing confidence in communicating. Whilst SLTs described goals to help with adjustment, other more practical forms of support were also coded to this category where SLTs were providing communication support to negotiate statutory entitlements and interviews. Malcomess describes assessment as determining the nature of the presenting problem [4]. Assessment represented 3.58% of goals. When analysing the data the coders added ‘review’ to the framework of goals as this activity is distinct from assessment and represented 4.31% of goals. ‘Activity to support therapy’ was also added as a goal category and represented 2.79% of goals.

**Table 3:** Therapy goals.

Goal category (level 1) Goal description (level 2)	Example (as described in the patient notes from which data were collected)	Number of goals	Percentage of goals (%)
**Assessment**	‘assess higher level language functions’	**59**	**3.58**
**Review**	‘review progress made in therapy’	**71**	**4.31**
**Rehabilitation (improving)**		**989**	**60.01**
Comprehension	‘improve auditory comprehension’	58	3.52
Expressive language	‘to produce longer/more complete verbal sentences’	273	16.57
Intelligibility	‘clearer speech’	15	0.91
Money skills	‘money handling skills’	14	0.85
Number skills	‘number recognition’	11	0.67
Phonological skills	‘phonological therapy’	36	2.18
Reading	‘identify functional written words’	142	8.62
Semantic skills	‘semantic categorisation of concrete items’	73	4.43
Time	‘following time’	1	0.06
Word finding	‘to be able to find words in conversation with more ease’	221	13.41
Writing	‘to be able to write short clear emails’	145	8.80
**Enabling**		**284**	**17.23**
AAC	‘Functional communication using low tech AAC’	30	1.82
Conversation support	‘Supported conversation using technology’	100	6.07
Participation in social conversation/activities	‘speak more fluently with golf friends’	19	1.15
Reading strategies	‘learn new reading strategies’	35	2.12
Return to work strategies	‘return to work strategies’	1	0.06
Total communication strategies	‘alternative ways to get message across’	28	1.70
Using everyday technology	‘use of spell check’	40	2.43
Word finding/self-cueing strategies	‘functional and compensatory strategies for word finding’	31	1.88
**Supportive**		**57**	**3.46**
Emotional support	‘exploring loss and gain’	9	0.55
Improve mood	‘to improve mood’	5	0.30
Increase confidence in communicating	‘to improve confidence in talking in group setting’	16	0.97
Managing frustration	‘frustration levels’	1	0.06
Providing information	‘to advise patient and family about impact and recovery from aphasia’	13	0.79
Support communication with other professionals/form completion	‘form filling support’	3	0.18
Support for family	‘communication support for family’	4	0.24
Vocational support	‘attend ‘fit for work’ interview’	6	0.36
**Activity to support therapy**		**46**	**2.79**
Discussing discharge	‘discharge planning’	5	0.30
Expert patient training	‘expert patient training’	2	0.12
Goal setting	‘to set goals for occupational therapy and speech therapy’	20	1.21
Handover	‘handover to new SLT’	4	0.24
Liaison with other staff/family	‘liaison with social worker’ ‘to demonstrate laptop comprehension tasks to family’	5	0.30
Preparing/monitoring homework	‘set up home exercises’	3	0.18
Therapy planning	‘establish motivation for therapy’	7	0.42
**Insufficient information**		**141**	**8.56**
Goal not sufficiently described	‘activity practice’, ‘to achieve 90% on tasks’	129	7.83
No goal recorded		12	0.73
**Not communication therapy**		**1**	**0.06%**
	**Grand Total**	**1648**	**100.00%**

**Fig 6:**
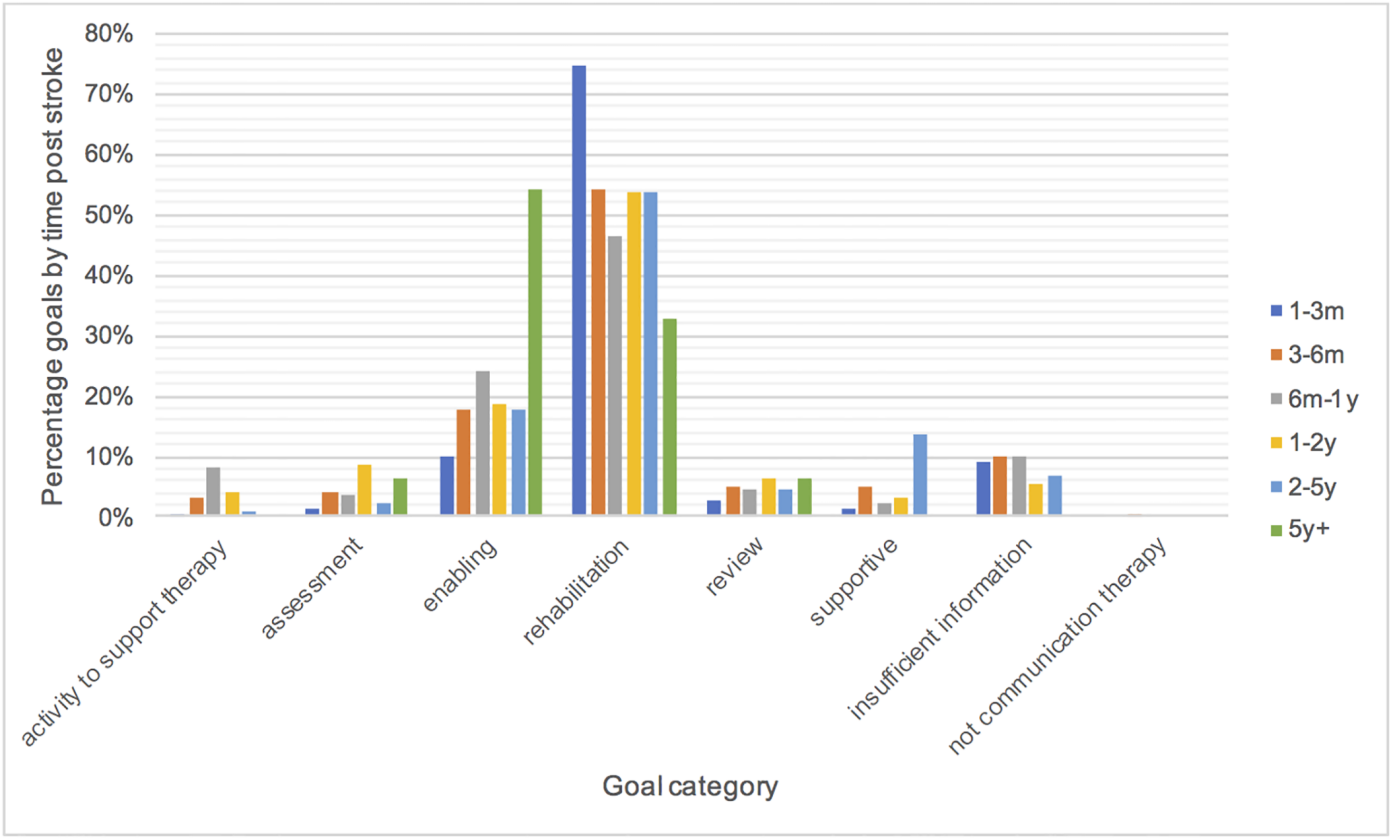
Therapy goals by time post stroke.

**Fig 7:**
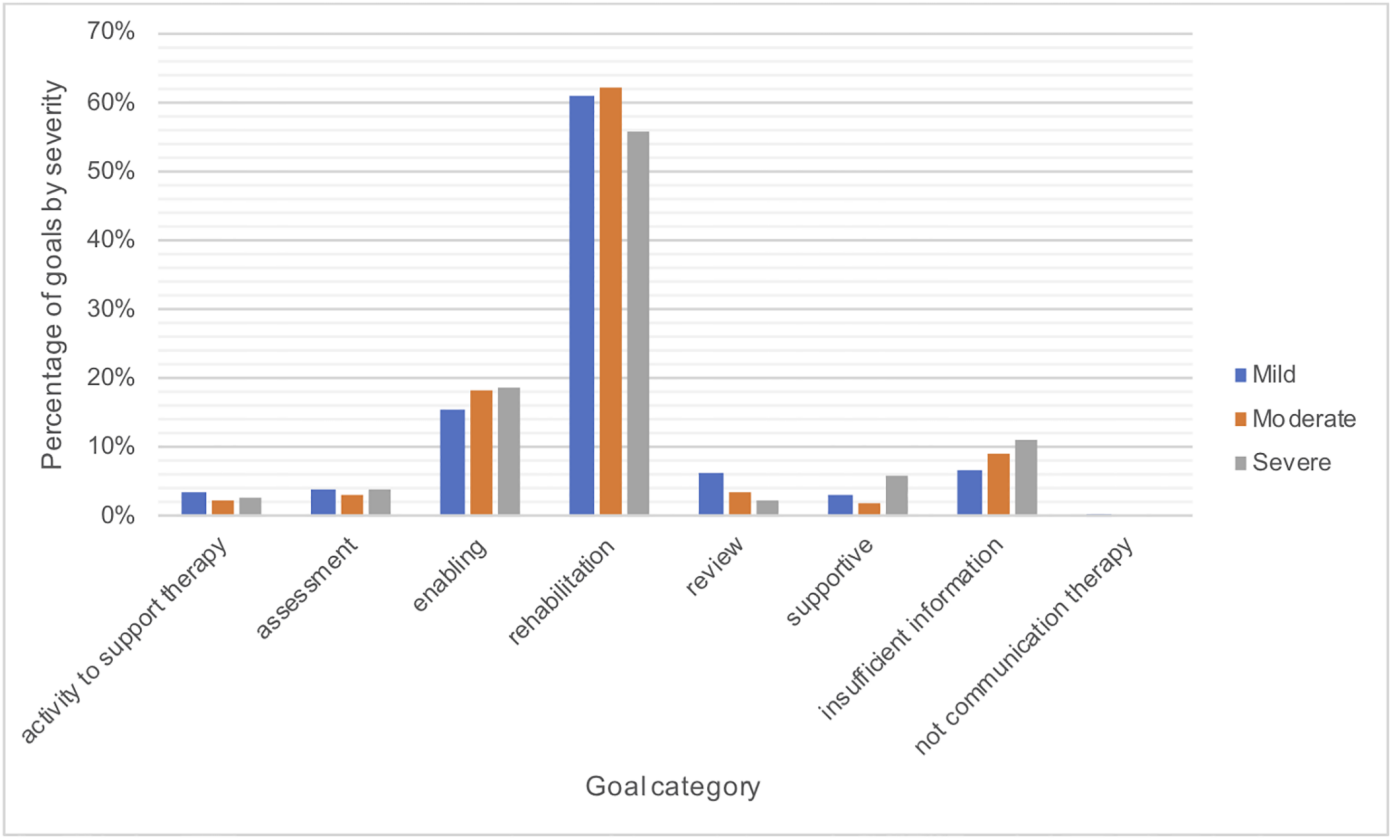
Therapy goals by severity of word finding.

**Fig 8:**
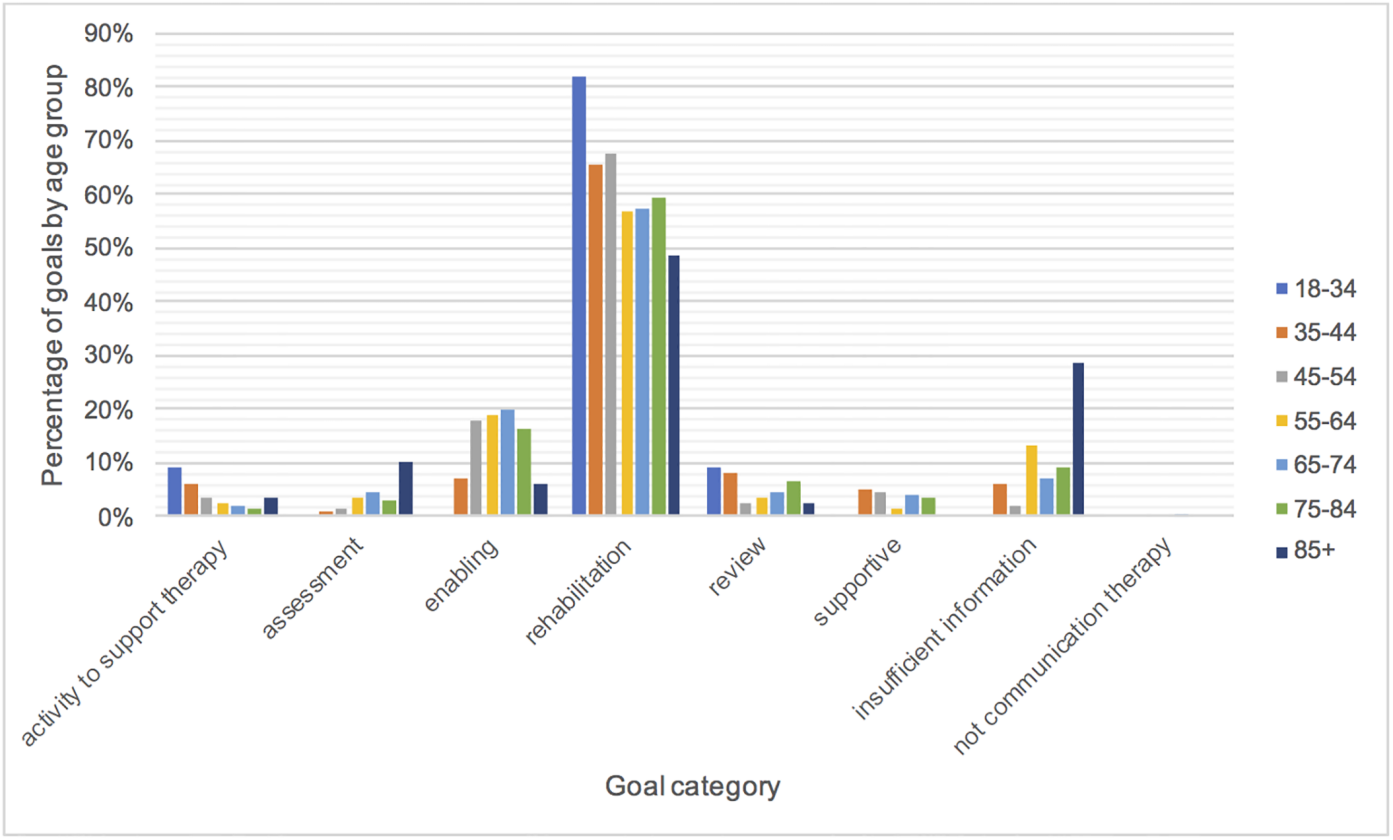
Therapy goals by age.

Figs 6–8 show the goals in each category as a percentage of the total number of goals in each time post stroke, severity and age group to see if there are any differences in the therapy focus for different subgroups. Fig 6 shows a trend towards provision of greater amounts of rehabilitation earlier post stroke (one-three months). Rehabilitation goals are more prevalent than enabling goals across time post stroke until five years. By five years post stroke the proportion of enabling goals is greater than the proportion of rehabilitation goals. The proportion of supportive goals also increased between two and five years post stroke. Differences between severities in Fig 7 are minimal. Fig 8 shows that rehabilitation goals decreased with increasing age. Activity to support therapy was slightly higher in the age groups below 44 years of age than in older age groups. Interestingly assessment was markedly more frequent for the over 85 year olds, however goals for this age group were particularly poorly described (almost 30% with insufficient information).

The majority of goals were split between rehabilitation and enabling for 19 of the 21 participating NHS trusts as expected. However, in two Trusts (C and R), no rehabilitation goals were recorded. In Trust C, 36.4% of goals focussed on the reviewing process, whilst 54.6% of goals were insufficiently described. In Trust R reviewing was again the most frequent goal (27%), with 15% assessment, 24% enabling and 24% insufficiently described.

### How much therapy is received?

The total number of therapy sessions received over the three month period observed and the number of therapy sessions per week was calculated (median averages, minimum and maximum sessions using data from 125 participants with recorded therapy sessions. The average number per week was calculated by dividing the total number by 12 assuming 12 weeks in a three month period). The median average number of sessions received in 3 months was 7 (minimum = 1, maximum = 41). The median average number of sessions per week was 0.58 (i.e. less than once a week) ranging from 0.08 to 3.42.

One hundred and twenty participants had the amount of time the therapy sessions lasted recorded. The median average total amount of therapy time received in the three month period of those participants with time recorded was 380 minutes (6.3 hours) ranging from 30 minutes to 2490 minutes (41.5 hours). The median average therapy time per week was 32 minutes (range: 2.5 minutes to 207.5 minutes (3.5 hours)). The median session length was 60 minutes (one hour) ranging from five minutes to 180 minutes (3 hours). Table 4 shows that the total therapy time received in a three month period goes down with time post stroke with those between one and three months post stroke receiving 11 hours of therapy at a frequency of almost one 60 minute session per week, whilst those more than five years post stroke received 2.75 hours of therapy in 45 minute sessions once a month on average. The older age group (85 years and above) received sessions of 45 minutes in length, slightly shorter than other age groups. There were no other notable differences between age groups and severities of word finding.

**Table 4:** Amount of therapy received by time since stroke, age and severity.

		Median number of sessions in 3 months	Median total therapy time in 3 months (mins)	Median length of sessions (mins)	Median frequency of therapy sessions per week
**Overall**		7	380	60	0.58
**Time since stroke**	1-3m	11.5	660	60	0.96
	3-6m	8	435	60	0.67
	6m-1y	6	345	50	0.50
	1-2y	5	307.5	60	0.42
	2-5y	4.5	245	60	0.38
	5y+	3	165	45	0.25
**Age group**	18–34	7	405	60	0.58
	35–44	9	645	60	0.75
	45–54	11	550	60	0.92
	55–64	7	330	60	0.58
	65–74	6	335	60	0.50
	75–84	6	345	50	0.50
	85+	8	400	45	0.67
**Severity**	Mild	7	372.5	60	0.58
	Moderate	6	357.5	60	0.50
	Severe	6.5	427.5	60	0.54

### Who delivers therapy and how is it delivered?

**Fig 9:**
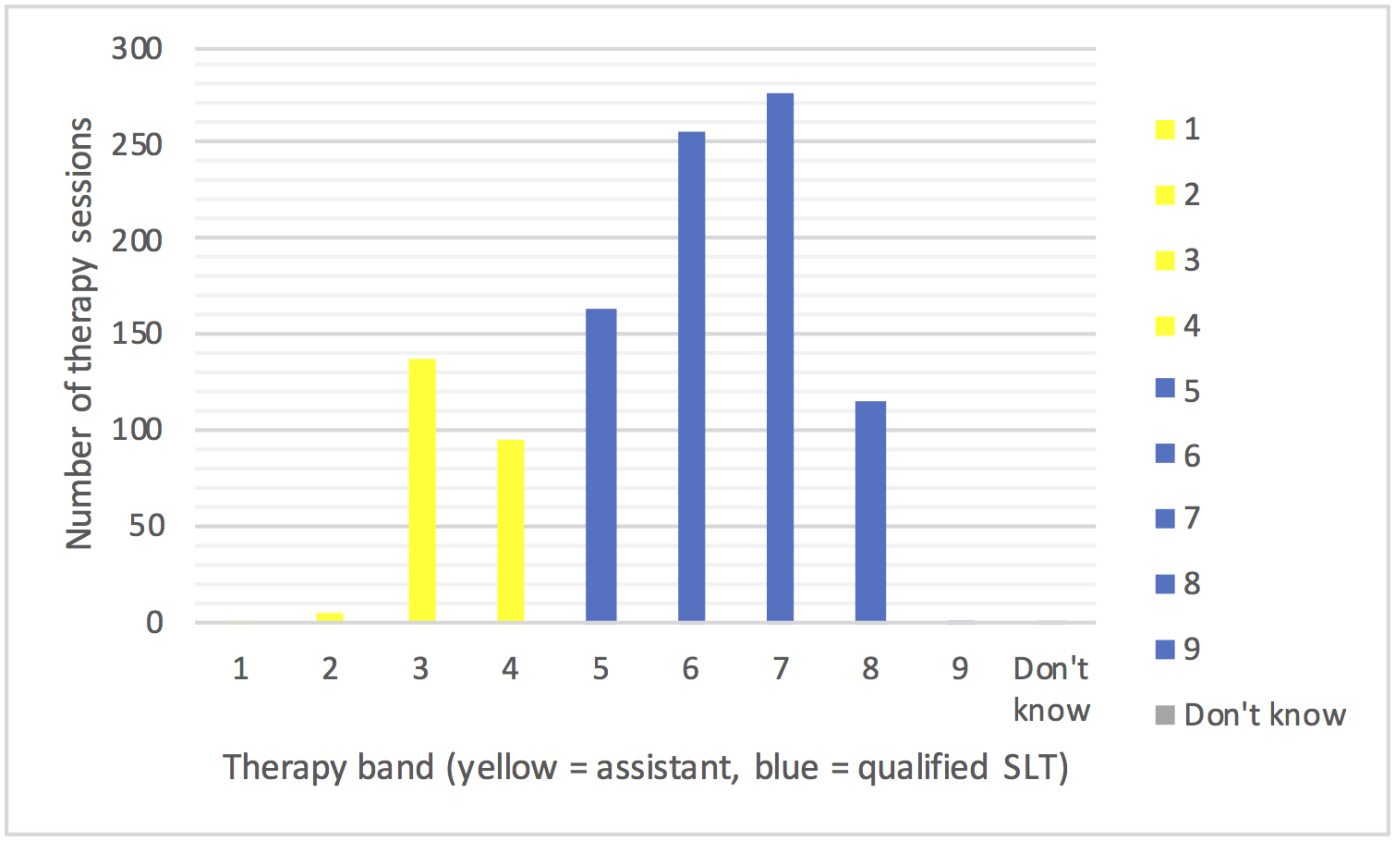
Number of therapy sessions delivered by qualified speech and language therapists and speech and language therapy assistants.

The majority of therapy sessions (77%) were delivered by qualified speech and language therapists with 23% of sessions being led by speech and language therapy assistants. In the UK, speech and language therapists and assistants working for the NHS are paid according to the ‘Agenda for Change’ pay scale. A higher band denotes a higher level of expertise. Fig 9 shows that the majority of therapy sessions were provided by qualified speech and language therapists at band six and seven. Assistant led sessions were mainly delivered by assistants on bands three and four.

Of the 1052 therapy sessions recorded, the vast majority, 945 (90%), were delivered in one to one sessions face to face. One hundred sessions (9.5%) were group sessions, with only one session described as using tele-health and five sessions using the telephone. On average face to face therapy sessions (group or one-to-one) required the therapist or patient to travel 13.2 miles, with therapists traveling up to 48 miles for some sessions.

## Discussion

This paper presents data on the therapy received by 278 people with aphasia across a range of NHS trusts, both urban and rural in all four nations of the UK, providing a representative sample of the therapy provided for community dwelling adults with post stroke aphasia. Participants were between one month and 36 years post stroke. In the three month time window prior to randomisation to the Big CACTUS study, 45% of these people were in receipt of speech and language therapy. The results show that people with aphasia are more likely to receive therapy than not if they are within one year of their stroke, but less likely to receive therapy if they are beyond a year post stroke despite evidence that people with aphasia can benefit from speech and language therapy in the chronic phase [5]. It is interesting that a lower proportion of people with mild word finding difficulties were in receipt of therapy than those with moderate and severe difficulties. It is possible that these participants are felt to function sufficiently well and resources are therefore focussed on those who have more difficulty in functional communication situations. If this is the case, it would be interesting to know whether decisions about the need for therapy are those of the people with aphasia themselves or those of the therapists. There was a wide range of participants in receipt of therapy across the 21 NHS trusts, from 20 to 91%. This may be due in part to the length of time post stroke the participants were in each area, however the data suggest that this cannot account for all of the differences in therapy provision thus indicating the presence of a ‘post code lottery’ for speech and language therapy after stroke in 2014–2016.

The content of therapy provided was analysed from information that was clinically recorded for each therapy session provided. More than 8.56 percent of the therapy goals were not sufficiently well described to be able to infer either the therapy goal or the focus of activity within a session. There was also variation in the way the content of therapy sessions was described with some clearly patient centred goals specified, some session activity descriptions recorded and some goals/descriptions of tasks that the therapists needed to complete. Where there was sufficient description of the focus of therapy sessions, the goals could be inferred. The Kate Malcomess Care Aims were used as a starting point for creating a framework within which to code the goals [4]. Assessment (3.58%), rehabilitation (60.01%), enabling (17.23%), and supportive (3.46%) were all Care Aim categories that goals could be aligned to. In addition, review (4.31%) and activity to support therapy (2.79%) were added as categories to capture the remaining goals. The majority of goals focussed on rehabilitation with the intention of improving language skills and reducing the impairment. The intention of working on the impairment was often to achieve a functional goal e.g. to be able to write short clear emails. This is consistent with the emphasis on functional approaches to therapy taken in the survey of Australian therapists [15]. The next most frequently described goals were categorised as enabling goals, helping to reduce the impact of the communication disorder in order to enable the person to participate. These often focussed on being able to engage in social, leisure and work activities consistent with one of the priorities for people with aphasia described by Worrall et al (2010) [2]. The other main intervention goal category was ‘supportive’. This included providing information which was identified as another priority for people with aphasia [2]. Therapists also provided practical support with communication tasks. The other type of support was emotional support and providing support for mood, confidence and frustration. These counselling type activities were also recognised by Australian therapists as an important part of the SLT role in community settings but with little training to provide them [15]. Therapy goals were not only about direct provision of therapy but also about work the therapists needed to do to ensure therapy was supported. Assessment of the language difficulties to direct therapy and review of progress towards goals was also an integral part of therapy provision.

An interesting trend was that rehabilitation was a greater focus of therapy at one to three months post stroke than at later time points and by five years enabling was more prevalent than rehabilitation. Rehabilitation also reduced with increasing age. It is not known whether this is due to therapist beliefs about who can benefit from rehabilitation, patients’ ability to participate in rehabilitation activities or choices about where to focus limited resources. It was noted that the over 85 age group were assessed more than other age groups but the purpose of therapy sessions was three times less well defined than for other age groups with almost 30% of goals for the over 85’s insufficiently described. This is an interesting finding worthy of further investigation. Is it more difficult to define useful goals? Are therapists concerned about what they might be able to achieve? Do increasing co-morbidities and frailty reduce the consistency of the requirements of therapy?

Whilst rehabilitation and enabling were the most prevalent goals in the majority of NHS trusts, two trusts focussed heavily on assessing and reviewing their patients with small amounts of enabling and support but no rehabilitation. Not only does this reinforce the notion of a post code lottery, but if little intervention is being provided, it raises the question of the purpose of assessing or reviewing patients.

The median number of therapy sessions received in the three month period of observation by these community dwelling people with aphasia was seven. The median average therapy time received was 6.3 hours in 60 minute sessions once every two weeks. These amounts of therapy could be considered sub therapeutic in terms of dose and intensity compared with evidence reported in the literature [7]. It must be noted however that participants may have had therapy in the weeks prior to the three month period observed and the weeks following which may lead to a greater dose if not intensity. Where resources are in short supply, therapists have the difficult ethical decision of providing therapeutic levels of intervention to some patients, or providing sub therapeutic levels to all. Currently it looks as if the latter is most common in practice.

The majority of speech and language therapy sessions were provided face to face and one to one by qualified speech and language therapists. Only 9.5% of therapy sessions were provided in a group. This is consistent with views of therapists in Australia that groups can be difficult to run as they are not always practical with differences in stage and progress and distances to travel [15]. In this paper, the average travel distance for groups was similar to those for one to one sessions at an average of just over 13 miles. Assistants were being used to deliver a third of therapy sessions, however other efficiency measures e.g. use of telehealth were not yet being embraced routinely in 2014–2016 despite travel distances of up to 48 miles taking up considerable amounts of therapist time.

### Limitations of the study

Although this study provides information from a large number, 278, of people with aphasia across 21 NHS trust in the UK, a minimum of 250,000 [24] people are likely to be living with aphasia in the UK therefore this sample is only 0.01% of the whole population. The sample is also likely to be influenced by where recruiting therapists identified patients from for inclusion in the Big CACTUS trial. Nine of the recruiting NHS SLT departments served predominantly rural populations, eight served a mix of rural and urban populations and only four served predominantly urban populations, skewing the population in this study towards a more rural population. It is possible that the provision of services to people with aphasia living in rural areas differs from that provided to people living in urban areas. A larger proportion of participants recruited to the Big CACTUS study were likely to have been in receipt of therapy than in the whole population of people living with aphasia as, although participants were identified from past patient records and voluntary sector groups as well as those on current case loads, many people longer term post stroke may not be known/still recorded on past lists or attend voluntary groups. In addition, the median time post stroke was two years in the sample which is likely to be less than in the whole population of people living long term with aphasia. We know from the data that therapy provision declines over time post stroke. There are therefore a number of reasons why the average amounts of therapy received in this sample may be higher than for the average population of people living with aphasia long term. It is always important to remember that people willing to be participants of a trial may differ in some way to the population they are selected to represent. The data is only as accurate as that recorded in patient therapy notes. In addition, sometimes limited descriptions had to be interpreted by the authors coding the data. This data provided a three month snap shot of what participants of the Big CACTUS study received as usual speech and language therapy care prior to being randomised to trial intervention groups. It is possible that some participants were in receipt of therapy a week before or after this window despite not receiving any during that three months. Also, it is unlikely that what was received in the three month snap shot was replicated for the next three months (i.e. it cannot be assumed that the amount received over a 6 month period would be double that recorded in the 3 month observed period). Results may therefore not always be a perfectly true reflection of the therapy received in practice in the UK.

## Conclusion

Less than half of people living with aphasia in the community are in receipt of speech and language therapy in a given three month time window. Those less than a year post stroke are more likely to be in receipt of therapy than those more than a year post stroke. Whilst a wide range of goals are pursued in therapy including assessment, review, rehabilitation, enabling, support and support for therapy activity, therapists most commonly work towards rehabilitation goals in the first five years after stroke, moving to predominantly enabling goals after five years. Given the intensity and dose shown in the literature to be required for good rehabilitation outcomes, the prevalence of rehabilitation goals is somewhat at odds with the amounts of therapy being received with the average dose being 6.3 hours for those in receipt of therapy over a three month period provided on average at a low intensity of one hour every two weeks. The majority of therapy sessions are provided face to face using a combination of qualified SLTs (two thirds) and SLT assistants (one third) but potentially more efficient options for delivering therapy including groups and telehealth are rarely used. A post code lottery has been observed for both the likelihood of receiving therapy as a community dwelling stroke survivor with aphasia and for the type of goal most likely to be the focus of therapy. In order to use resources wisely to optimise outcomes from therapy for all there is a need to ensure the dose and intensity of treatment provided are in line with the type of goals being pursued. To achieve this, it is crucial that innovative ways of providing therapy efficiently become integral to routine clinical practice.

## Supporting information

S1 File Participant demographics. (CSV)

S2 File Unique therapy goals. (CSV)

S3 File Goals per participant. (CSV)
